# *In vitro* determinants of asbestos fiber toxicity: effect on the relative toxicity of Libby amphibole in primary human airway epithelial cells

**DOI:** 10.1186/1743-8977-11-2

**Published:** 2014-01-08

**Authors:** Kelly E Duncan, Philip M Cook, Stephen H Gavett, Lisa A Dailey, Ron K Mahoney, Andrew J Ghio, Victor L Roggli, Robert B Devlin

**Affiliations:** 1 Cancer Biology Program, Fox Chase Cancer Center, Philadelphia, PA 19111, USA; 2 Mid-Continent Ecology Division, National Health and Environmental Effects Research Laboratory, U.S. Environmental Protection Agency, Duluth, MN 55804, USA; 3 Environmental Public Health Division, National Health and Environmental Effects Research Laboratory, U.S. Environmental Protection Agency, Research Triangle Park, NC 27711, USA; 4 EMSL Analytical, Inc., Libby, MT 59923, USA; 5 Department of Pathology, Duke University Medical Center, Duke University, Durham, NC 27710, USA

**Keywords:** Libby amphibole, Airway epithelium, Relative toxicity, Inflammation, Interleukin-8, Dose metrics

## Abstract

**Background:**

An abnormally high incidence of lung disease has been observed in the residents of Libby, Montana, which has been attributed to occupational and environmental exposure to fibrous amphiboles originating from a nearby contaminated vermiculite mine. The composition of Libby amphibole (LA) is complex and minimal toxicity data are available. In this study, we conduct a comparative particle toxicity analysis of LA compared with standard reference asbestiform amphibole samples.

**Methods:**

Primary human airway epithelial cells (HAEC) were exposed to two different LA samples as well as standard amphibole reference samples. Analysis of the samples included a complete particle size distribution analysis, calculation of surface area by electron microscopy and by gas adsorption and quantification of surface-conjugated iron and hydroxyl radical production by the fibers. Interleukin-8 mRNA levels were quantified by qRT-PCR to measure relative pro-inflammatory response induced in HAEC in response to amphibole fiber exposure. The relative contribution of key physicochemical determinants on the observed pro-inflammatory response were also evaluated.

**Results:**

The RTI amosite reference sample contained the longest fibers and demonstrated the greatest potency at increasing IL-8 transcript levels when evaluated on an equal mass basis. The two LA samples and the UICC amosite reference sample consisted of similar particle numbers per milligram as well as similar particle size distributions and induced comparable levels of IL-8 mRNA. A strong correlation was observed between the elongated particle (aspect ratio ≥3:1) dose metrics of length and external surface area. Expression of the IL-8 data with respect to either of these metrics eliminated the differential response between the RTI amosite sample and the other samples that was observed when HAEC were exposed on an equal mass basis.

**Conclusions:**

On an equal mass basis, LA is as potent as the UICC amosite reference sample at inducing a pro-inflammatory response in HAEC but is less potent than the RTI amosite sample. The results of this study show that the particle length and particle surface area are highly correlated metrics that contribute significantly to the toxicological potential of these amphibole samples with respect to the inflammogenic response induced in airway epithelial cells.

## Background

The vermiculite deposit located in the Rainy Creek Complex near Libby, Montana produced eighty percent of the world’s supply of vermiculite from the 1920’s until its closure in 1990 when it was discovered that it was contaminated with naturally-occurring asbestiform fibers [1]. Epidemiology studies conducted since that time have reported abnormally high incidences of asbestos-related lung diseases, including mesothelioma, lung cancer and asbestosis in the Libby population, which continue to impact them even today [2-7]. Moreover, the vermiculite originating from the Libby mine during its operational years was shipped all over the world and used as insulation in hundreds of thousands of homes and as an additive in gardening soil, potentially impacting the health of millions of individuals [1]. Libby, MT became a designated Superfund site in 1999 leading to extensive cleanup and remediation and in 2009 the U.S. Environmental Protection Agency announced a public health emergency status for the community.

The respirable fraction of the amphibole fibers obtained from the vermiculite deposit, termed Libby amphibole (LA), has been extensively characterized by the U.S. Geological Survey and shown to consist primarily of the non-regulated amphibole varieties winchite (83%) and richterite (11%) and a small percentage of the regulated amphibole tremolite (6%) [8]. Minimal, if any, toxicological and risk assessment information exists for these non-regulated forms of asbestiform minerals, especially when they are present as a complex mixture. Consequently, a multi-disciplinary cross-organizational workgroup was created and led by the U.S. EPA to address datagaps regarding the toxicity of LA that would subsequently be used to inform the risk assessment for this complex amphibole mixture [9]. One component of the Libby Action Plan (LAP) involves a comparative toxicity assessment both *in vitro* in cultured cells and *in vivo* in animals in which LA is compared to standard reference amphibole samples that have extensive toxicological and risk assessment information available. Thus, the current study reports on the findings of the *in vitro* comparative toxicity analysis of Libby amphibole against two different reference samples of amosite, which will complement the rat inhalation and instillation studies conducted on these same LA samples as part of the LAP.

Cultured primary human airway epithelial cells (HAEC) sampled from the bronchi were exposed in this study to two samples of LA collected in the years 2000 and 2007 as well as two reference samples of amosite and the relative pro-inflammatory response was evaluated by measuring mRNA transcript levels of genes that are known to be involved in mounting an inflammatory response: the cytokines interleukin-8 (IL-8), interleukin-6 (IL-6), tumor necrosis factor (TNF), and cyclooxygenase-2 (COX2) - the gene coding for a key component of the PGE2 pathway. HAEC were selected for this study since this cell type populates the conducting airways down to the terminal bronchiole region and consequently is the first target tissue encountered by inhaled noxious agents such as fibers from asbestos [10]. Moreover, HAEC are known to be significant producers of several pro-inflammatory mediators and growth factors that can contribute to the development of fibrotic or neoplastic lesions in the lung following chronic or high dose acute exposures to airborne fibers from asbestos [10-13]. Of the four pro-inflammatory biomarkers analyzed, we focused special attention on IL-8, a chemokine that has demonstrated a highly robust response in airway epithelial cells both *in vitro* and *in vivo* upon exposure to a diverse set of inhaled particles and gases, including fibers from asbestos [12,14-20]. This chemokine, which is known to attract neutrophils into the lung has been shown to be elevated in induced sputum and serum samples from patients diagnosed with asbestosis [21], a form of interstitial pulmonary fibrosis caused by the inhalation of asbestos fibers that is characterized by a persistent neutrophilic inflammatory response in the airways [22]. Due to the importance of IL-8 in the initiation and persistence of neutrophilic inflammation *in vivo* this chemokine was chosen in this study for the comparative metric for the determination of relative toxicity of the amphibole samples.

To fully understand the toxicological potential of LA, we additionally present in this report a comprehensive characterization of the physicochemical properties of the two LA samples, which have not been reported to date, as well as for the two amosite samples to supplement the existing literature. We further evaluate the effect of particle number, surface area, particle size distribution and reactive oxygen species production on the IL-8 response in an attempt to understand what physicochemical properties are critical to the toxicity of these asbestiform fibers.

## Results

### Comparison of amphibole physical properties

**Figure 1:**
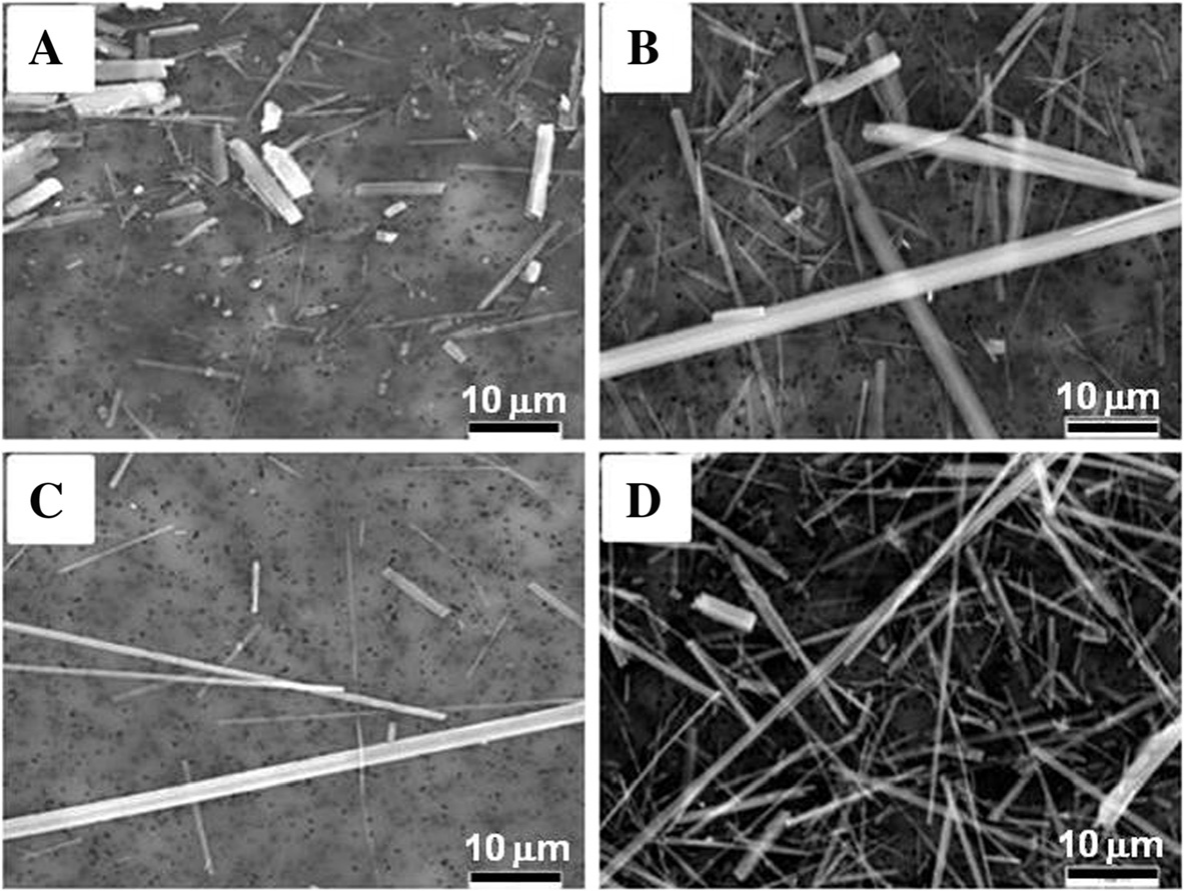
**Scanning electron microscopy images of amphibole samples.** Representative SEM images obtained at 2000x magnification of **(A)** LA2000, **(B)** LA2007, **(C)** RTI amosite and **(D)** UICC amosite illustrating the relative morphology and size distribution of the mineral particles. Images do not reflect equal masses of each sample. Scale bars are 10 μm.

Figure 1 shows representative SEM images obtained at 2000x magnification of the two site-specific LA samples, referred to herein as LA2000 (collected in the year 2000) and LA2007 (collected in the year 2007), respectively, as well as the two standard reference samples of amosite (RTI and UICC). Although the sample masses shown differ, visible differences in particle size distribution (PSD) and morphology are apparent among the samples; specifically the presence of blocky particles in conjunction with very thin fibers in the LA2000 sample (Figure 1A) as compared to the presence of some very long fibers in the other amphibole samples particularly the RTI amosite sample (Figure 1C).

TEM analysis of each amphibole sample was conducted by counting approximately 500 mineral particles with a minimum length of 0.2 μm to accurately quantify the PSD through the measurement of the length, width and aspect ratio of each counted particle. All mineral types were included in this analysis such that both amphibole and non-amphibole particles were counted. Complete PSD tables for each sample are detailed in Additional file 1: Tables S1-S4 and are summarized in Table 1. A review of the TEM summary statistics shows that the LA2000, LA2007 and UICC amosite samples contain similar numbers of total particles per milligram whereas the RTI amosite sample contains significantly fewer total particles at approximately 10-fold less per milligram of sample than the other three samples. The LA2000 sample contains significantly more elongated mineral particles (EMP) with an aspect ratio of ≥ 3:1 with 80% of the counted particles meeting this criterion, whereas the LA2007 and RTI amosite samples are composed of ~50% EMP and the UICC amosite sample only 34% EMP.

**Table 1:** Characterization of amphibole samples

	**LA2000**	**LA2007**	**RTI amosite**	**UICC amosite**
**Particle count**				
*N* (Total Particles)	561	510	588	525
*N* (EMP)^*a*^	450	250	292	178
**Particle number /mg**				
Total particles x10^7^ /mg	98.2	103	9.15	94.2
EMP ^*a*^ x10^7^ /mg	78.7	50.5	4.5	31.9
**Particle size distribution**				
Total particle mean length (μm)	3.7 ± 0.2	2.3 ± 0.2	6.4 ± 0.6	2.1 ± 0.3
Total particle mean width (μm)	0.36 ± 0.02	0.36 ± 0.01	0.44 ± 0.01	0.43 ± 0.01
Total particle mean aspect ratio	12.8 ± 0.6	8.4 ± 0.7	16.9 ± 1.6	5.6 ± 0.6
EMP ^*a*^ mean length (μm)	4.4 ± 0.2	3.8 ± 0.3	12.1 ± 1.2	4.3 ± 0.5
EMP ^*a*^ mean width (μm)	0.30 ± 0.01	0.29 ± 0.02	0.37 ± 0.01	0.27 ± 0.01
EMP ^*a*^ mean aspect ratio	15.5 ± 0.6	15.1 ± 1.2	32.4 ± 3.0	13.0 ± 1.0
**Surface area**				
Total surface area by GA (m^2^/g)^*b*^	5.3	7.4	3.1	4.8
EMP ^*a*^ surface area by TEM (m^2^/g)^*c*^	1.1	2.6	2.8	1.5
**Sum of particle length**				
∑ (All particle lengths) x10^6^ (μm/μg)	1.02	2.89	2.15	1.68
∑ ( EMP ^*a*^ lengths) x10^6^ (μm/μg )	0.97	2.39	2.03	1.20

^*a*^ Elongated mineral particle (EMP) defined as having aspect ratio ≥ 3:1.

^*b*^ Measured by Kr gas adsorption (GA) and BET analysis.

^*c*^ Measured by TEM and calculated using the equation SA = [L*W + L*T + W*T]/[L*W*T*ρ] – See Methods section for detailed description.

It is unlikely that the 10-fold difference in total particle number between the RTI amosite sample and the other three amphibole samples is due to differences in particle density, but rather because the RTI amosite particles are longer and/or thicker. Indeed, Table 1 shows that the average length and aspect ratio of RTI amosite particles is greater than that of the other three samples and the mean width is greater than that of either LA sample and greater than the UICC amosite sample when only EMP are considered. The mean length, width and aspect ratio for the two LA samples and the UICC amosite samples are fairly comparable when only EMP are included; however the mean lengths are reduced for the LA2007 and UICC amosite samples when all particles are counted, which is attributed to the greater percentage of non-EMP particles in these samples.

**Figure 2:**
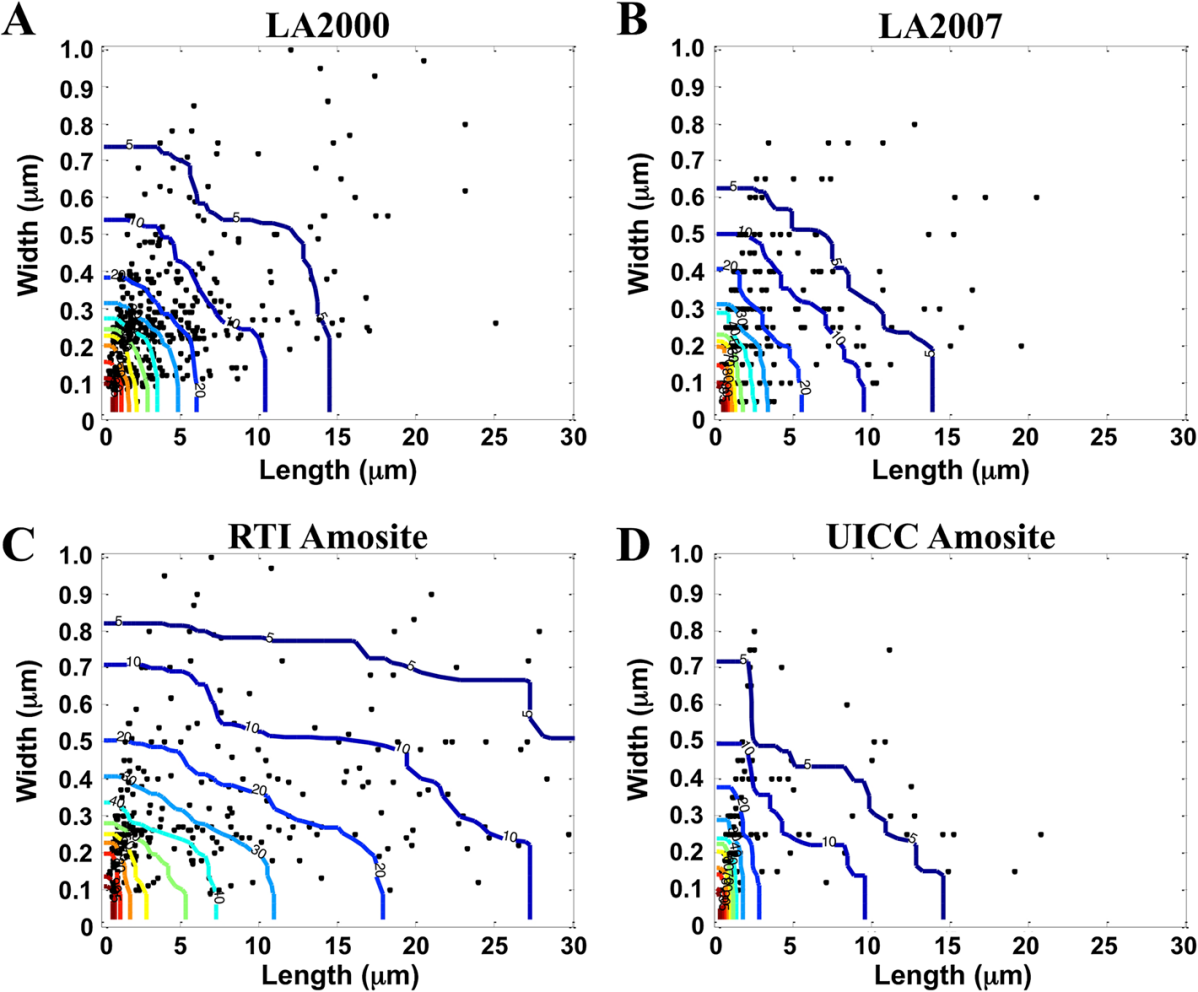
**Particle size distribution contour plots of the elongated mineral particles within each amphibole sample.** Each dot represents the respective length (y-axis) and width (x-axis) dimensions for each EMP having an aspect ratio ≥ 3:1 counted in each amphibole sample. Panel **A** shows data from LA2000; Panel **B** shows data from LA2007; Panel **C** shows data from RTI Amosite; Panel **D** shows data from UICC Amosite. Total number of EMP equals 450 (LA2000), 250 (LA2007), 292 (RTI amosite), 178 (UICC amosite). Only those particles with L ≤ 30 μm and W ≤ 1.0 μm are presented. Colored contour lines represent the L and W dimensions that correspond with a specific percentile cutoff of particle size distribution ranging from 95^th^ percentile (dark red) to 1^st^ percentile (dark blue). Percentiles are indicative of the percentage of EMP that fall to the right of the contour line.

The measured length by width particle distribution contour scatter plots presented in Figure 2 further illustrate the differences/similarities in PSD between the four amphibole samples. Each dot on the scatter plot represents a single EMP with AR ≥ 3:1 such that the position of the dot corresponds to the particle’s L and W value on the x- and y-axis, respectively, as determined by TEM. The colored contour lines represent the dimensional cutoff for a given percentile of particles in the PSD that are greater than the L and W values defined by the contour line. As an example, the 5^th^ percentile contour line indicates that at any L, W dimension point on the line, 5% of the EMPs are greater than or equal to the particle L, W size defined by that point on the contour line. Contour lines are shown starting from the 95^th^ percentile (dark red line closest to the origin) to the 5^th^ percentile (dark blue line). Comparison of the PSD contour plots for the two LA samples shows a high degree of similarity (Figure 2A and B), confirming that preparation of the more recently acquired LA2007 sample indeed resulted in a sample comparable to the LA2000 sample as intended [23]. The PSD obtained for the UICC amosite sample are comparable to the two LA samples whereas the PSD for the RTI amosite sample reflects the presence of much longer and thicker particles as compared to the other three samples, which is apparent in the summary statistics presented in Table 1.

Evaluation of the total surface area (TSA) of each sample as measured by gas adsorption shows that the sum of all particles SA in the LA2007 sample is slightly greater than that for the LA2000 sample, and both LA samples have more gas adsorbing SA than the reference amosite samples (Table 1). The results obtained by gas adsorption reflect the TSA of all particles in the sample regardless of mineralogical origin or morphology. To evaluate the relative SA of the elongated mineral particles with AR ≥ 3:1 only, SA values were subsequently calculated using the TEM data acquired for each sample as described in detail in the Materials and Methods section. Overall, the SA values for the EMP were lower than those calculated by gas adsorption as would be expected upon removing the contribution of the very small and platy particles from the SA calculation. Comparatively, the RTI amosite sample had the highest EMP SA while the LA2000 sample had the lowest EMP SA.

The length distribution of EMPs, including asbestiform fibers, has historically been an important metric for toxicity such that longer fibers are considered to be more pathogenic than shorter fibers. The implication is that the relationship between fiber length and potency is a continuum and that no specific length of fiber will adequately define a threshold for pathogenicity. Consequently, fiber length dose was calculated in the present study by summing the lengths of the particles and relating it to the mass of the sample counted on the filter. This provides length-specific proportional dose weighting to account for each particle’s hypothetical contribution to sample relative potency. Detailed in Table 1 are the results of the mass adjusted sum of the lengths dose calculated for all particles in the sample as well as for the EMP fraction (L/W aspect ratio ≥ 3.0). In both cases, the LA2007 and RTI amosite samples have the largest sum of the lengths dose and the LA2000 and UICC amosite samples have the lowest doses. Since the surface area of elongated particles which have modest relative differences in width is driven primarily by the length dimension of the EMPs it is not unexpected that the EMP sum of the length dose metric is highly correlated with the EMP surface area determined by TEM (*r* = 0.94).

### Cytotoxicity assay

**Figure 3:**
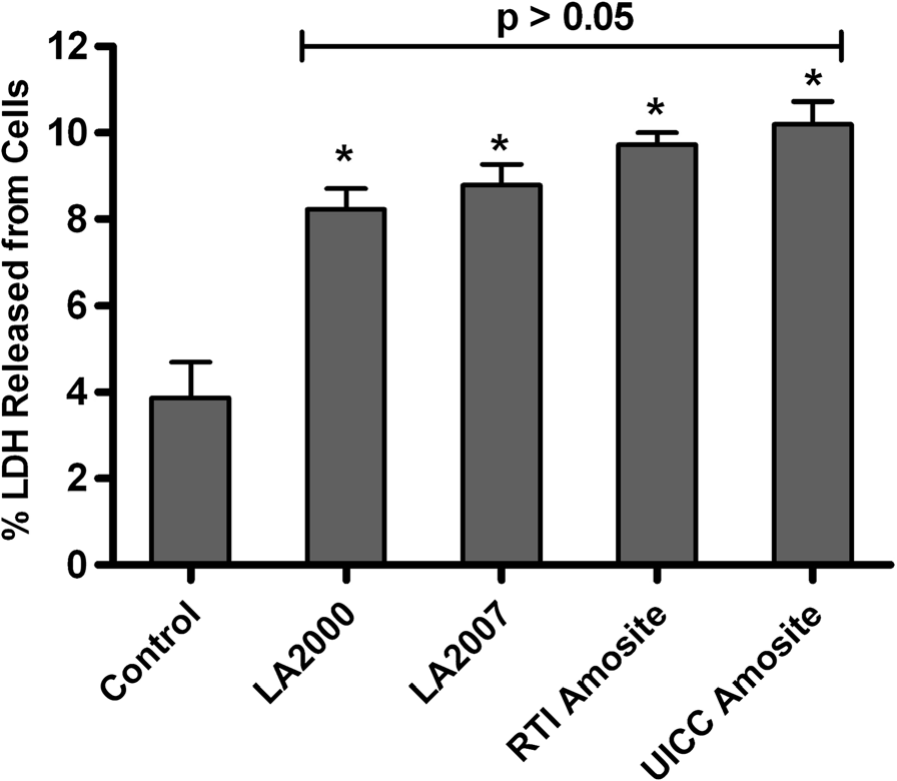
**Cytotoxicity of airway epithelial cells in response to exposure to the different amphibole samples.** Percentage of lactate dehydrogenase (LDH) leakage into the supernatant following a 24 h exposure to the highest dose (26.4 μg/cm^2^) of amphibole particles. * = Significant difference between treatment and no-treatment control. Difference is deemed significant when *p* < 0.05.

Primary human airway epithelial cells (HAEC) were exposed for 24 h to increasing mass doses (2.64, 13.2 or 26.4 μg/cm^2^) of each of the amphibole samples. Cellular cytotoxicity was assessed at the maximum dose of 26.4 μg/cm^2^ of each of the amphibole samples through measurement of cellular release of lactate dehydrogenase (LDH) into the cell culture medium. Shown in Figure 3 are the results of the cytotoxicity assay expressed as a percentage of LDH detected in the cell culture medium relative to the total LDH present in the cell lysates and supernatant combined. The unexposed control cells were found to have 3.9% LDH present in the supernatant whereas the exposed cells were found to have between 8% and 10% LDH in the supernatant. All samples exhibited a statistically-significant increase in LDH with respect to the no-treatment control (*p* < 0.05); however the percentage of LDH in the supernatant was ≤ 10% of the total cellular LDH for the amphibole-exposed cells confirming that the highest dose of amphibole particles used in this study is, at most, only mildly cytotoxic to the airway epithelial cells following exposures lasting up to 24 h. Notably, no statistically-significant difference (*p* > 0.05) was detected among the different amphibole samples with respect to LDH release indicating that the LA samples are no more cytotoxic on an equal mass basis than the reference samples of amosite.

### Pro-inflammatory response in HAEC exposed to amphibole samples

**Figure 4:**
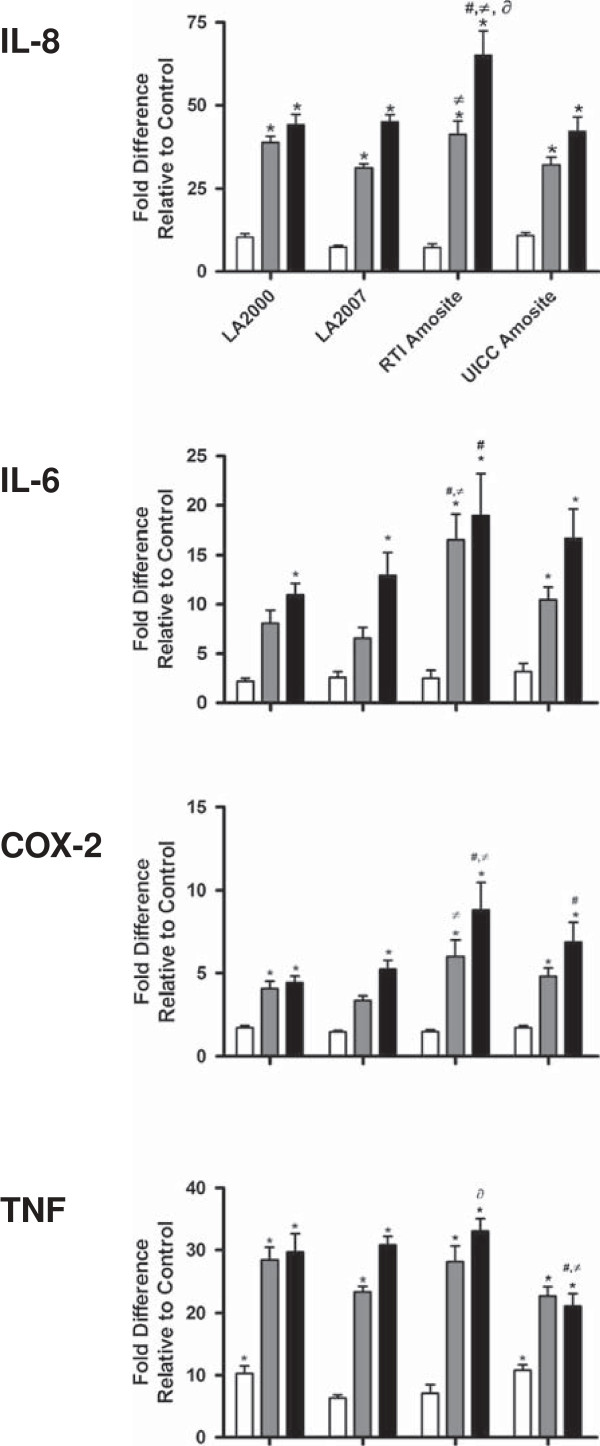
**qRT-PCR gene expression of select pro-inflammatory genes in HAEC in response to Libby amphibole with comparison to the standard reference amosite samples dosed on an equal mass basis.** Relative gene expression of the pro-inflammatory mediators *TNF* (TNF-a), *IL-6, PTGS2* (COX-2) and *IL8* measured by qRT-PCR following a 24 h exposure of HAEC to increasing doses of the four amphibole samples. HAEC were exposed on an equal mass basis to 2.64 (white bars), 13.2 (gray bars) or 26.4 (black bars) mg per cellular surface area (mg/cm^2^) to each of the amphibole samples. Data are presented as means ± SEM and are expressed as fold increase with respect to the vehicle-only control. Significant increases (*p* < 0.05) in mRNA transcript with respect to the vehicle-only control are denoted with an asterisk (*). Significant difference in response (*p* < 0.05) from LA2000 (#), LA2007 (≠), UICC amosite (∂).

Figure 4 shows the relative levels of mRNA coding for IL-8, TNF, IL-6, and COX2 quantified by qRT-PCR and expressed as fold change relative to the untreated control for each amphibole sample. The two higher doses (13.2 and 26.4 μg/cm^2^) induced statistically-significant (*p* < 0.05) increases in transcript levels of all genes and for all amphibole types relative to the untreated control. Both of the LA samples resulted in a robust and relatively equal response with respect to transcript levels from all four genes with fold increases ranging from 4-5 fold for COX-2 up to 40-50-fold for IL-8 over the untreated control at the maximum dose of 26.4 μg/cm^2^. No statistically-significant difference (*p* > 0.05) was observed in any biomarker response between the two LA samples or between either of the LA samples and the UICC amosite sample when compared on an equal mass basis. The RTI amosite sample was the most potent of all the amphibole samples at inducing a pro-inflammatory response in HAEC, with IL-8, IL-6, and COX-2 mRNA changes significantly elevated by the RTI sample compared with changes induced by either Libby sample. Additionally, IL-8 message was also statistically increased significantly more by the RTI than the UICC amosite sample.

**Figure 5:**
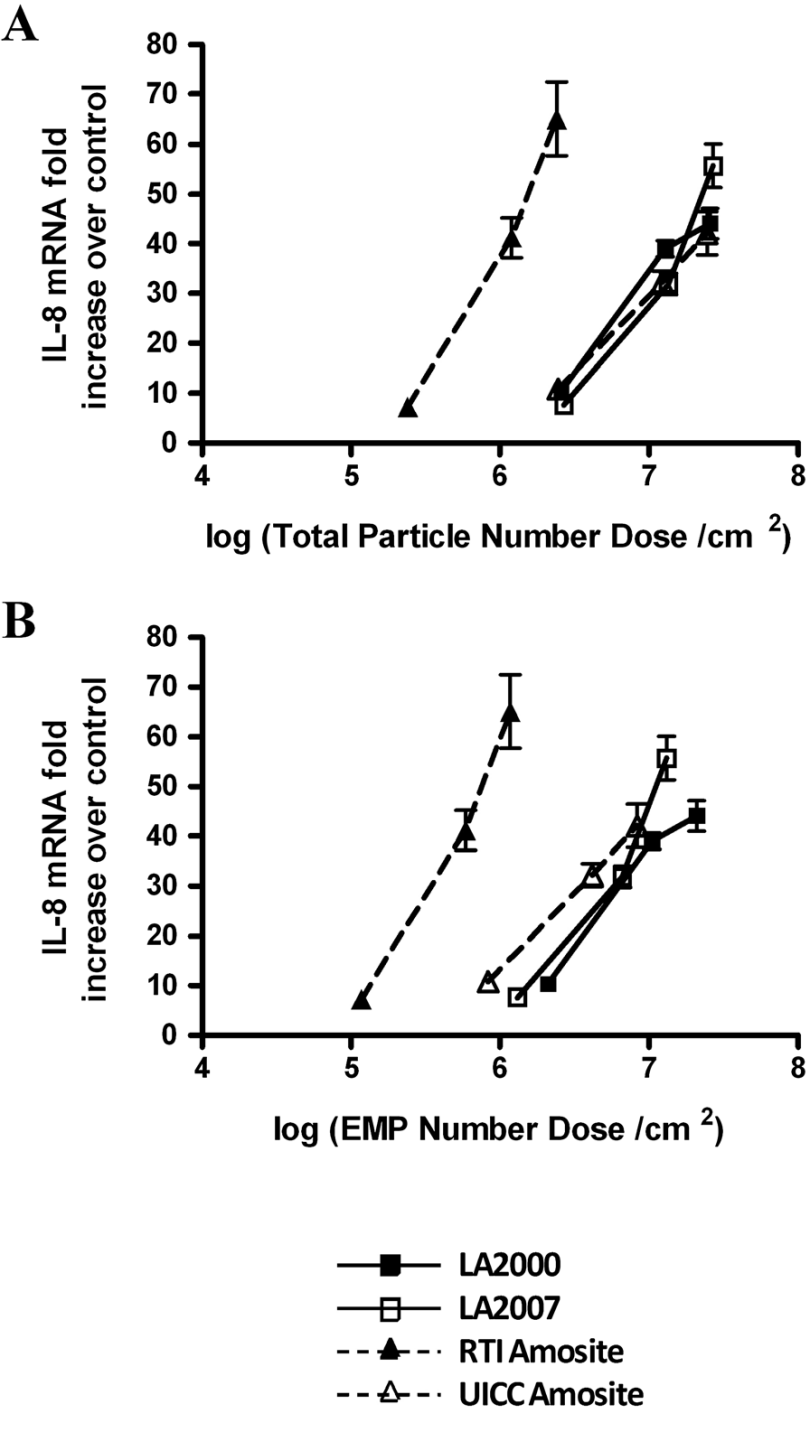
**Recalculation of the IL-8 gene expression data for particle number.** Quantitative RT-PCR gene expression data for IL-8 collected following a 24 h exposure of HAEC to increasing doses of the amphibole samples plotted with respect to **(A)** total particle number dose expressed as the log of the total number of mineral particles per cm^2^ cellular surface area and **(B)** elongated mineral particle (EMP) dose expressed as the log of the number of mineral particles with aspect ratio ≥ 3:1 per cm^2^ cellular surface area.

**Figure 6:**
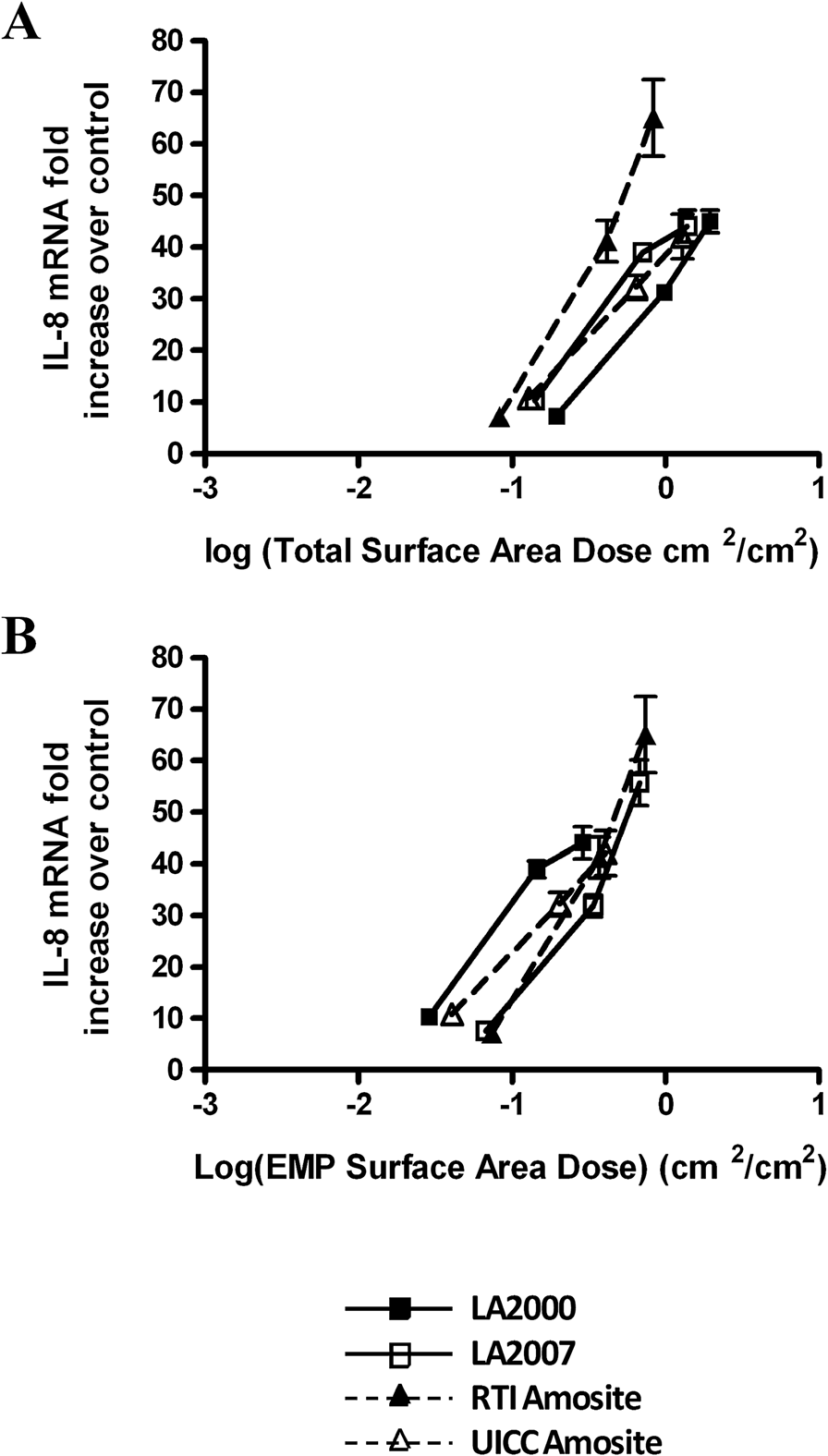
**Recalculation of the IL-8 gene expression data for particle surface area.** Quantitative RT-PCR gene expression data for IL-8 collected following a 24 h exposure of HAEC to increasing doses of the amphibole samples plotted with respect to **(A)** total surface area dose determined by gas adsorption and BET theory expressed as the log of the total surface area of the mineral particles per cellular surface area in units of cm^2^/cm^2^ and **(B)** elongated mineral particle (EMP) surface area calculated using transmission electron microscopy (TEM) expressed as the log of the surface area of particles with aspect ratio ≥ 3:1 per cellular surface area in units of cm^2^/cm^2^.

**Figure 7:**
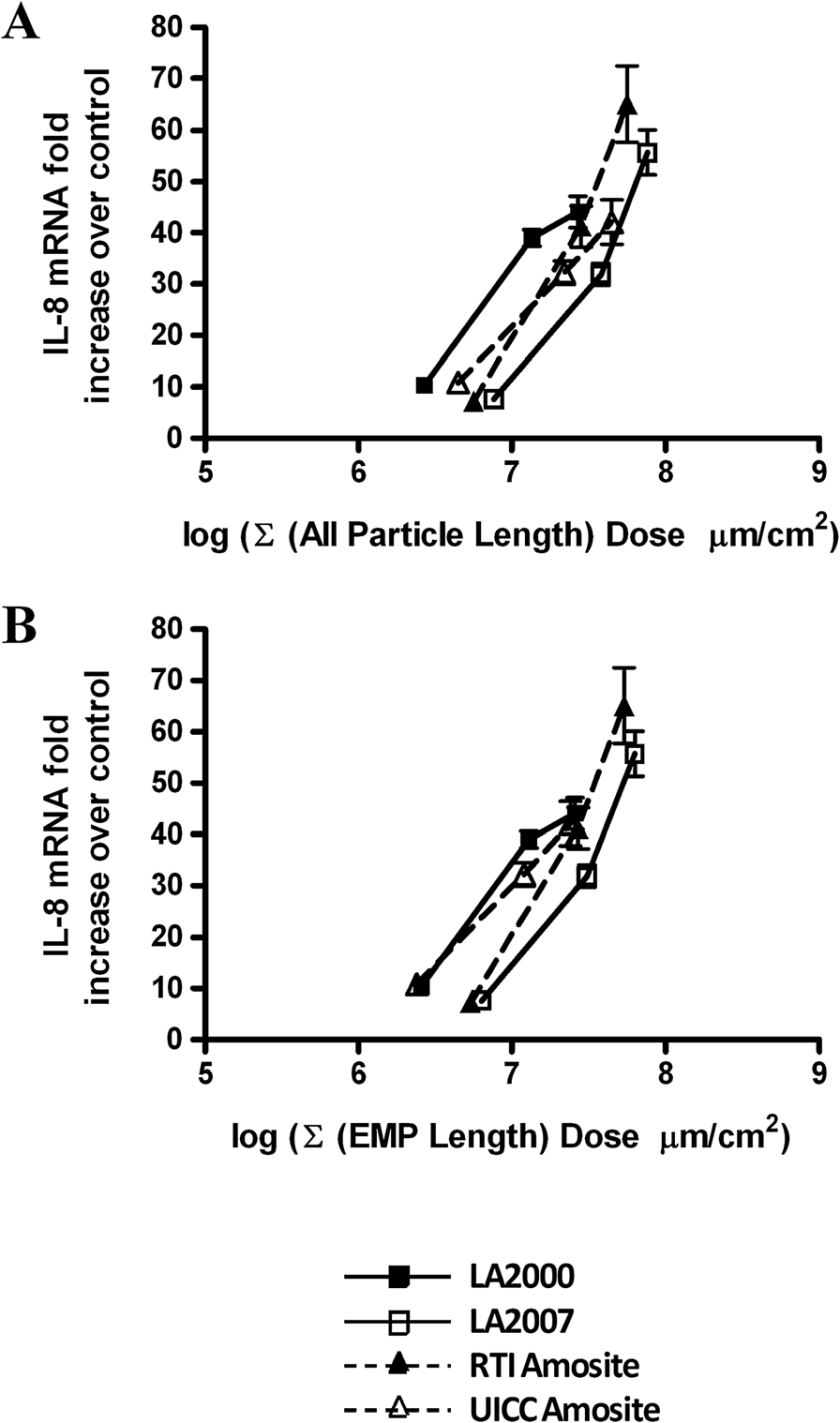
**Recalculation of the IL-8 gene expression data for the sum of particle lengths.** Quantitative RT-PCR gene expression data for IL-8 collected following a 24 h exposure of HAEC to increasing doses of the amphibole samples plotted with respect to **(A)** sum of the particle lengths for all particles expressed as the log of the ∑ (All Particle Lengths) per cellular surface area in units of μm/cm^2^ and **(B)** sum of the particle lengths for elongated mineral particles (EMP) only expressed as the log of the ∑ (EMP Lengths) with aspect ratio ≥ 3:1 per cellular surface area in units of μm/cm^2^.

Therefore we chose to use IL-8 to explore which metric (e.g. differences in particle number, surface area, and particle size distribution among the samples) might be responsible for the differential response to these asbestos samples. Figures 5, 6 and 7 show the same cellular response data as Figure 4, but the dose is re-calculated on the basis of total particle number (Figure 5A), elongated mineral particle number (Figure 5B), total surface area as measured by gas adsorption (Figure 6A), EMP-specific surface area as determined by TEM (Figure 6B), sum of the lengths dose for total particles (Figure 7A) and sum of the lengths dose for elongated mineral particles only (Figure 7B). The mass doses used in this study spanned a large enough range that, in many cases, equivalent doses were in fact administered with respect to these alternative metrics allowing for a direct comparison of the resultant cellular response data between the different amphibole samples.

Recalculation of the HAEC IL-8 response data as it relates to total particle number (Figure 5A) demonstrates equal potency between the LA2000, LA2007 and UICC amosite samples on a per particle basis; however, each particle from the RTI amosite sample is significantly more potent than all other samples at increasing IL-8 transcript levels in HAEC. Indeed, the highest dose of the RTI amosite sample, which contains the same number of total particles as the lowest dose of the LA2000, LA2007 and UICC amosite samples, induced an approximately 7-fold greater response in IL-8 gene expression. So although it may appear that total particle number may be an important determinant for the increase of IL-8 message based on the similarity in response between the two LA samples and the UICC amosite sample, the difference in response observed for the RTI amosite sample contradicts this association.

The elongated nature of asbestiform fibers has been shown to be critical to the mechanism of action of these minerals. If only EMP with AR ≥ 3:1 are significant contributors to the induced pro-inflammatory response in HAEC, then expressing the dose with respect to EMP only may provide a more relevant assessment of the relative potency of the different amphibole particle types. Figure 5B shows that the RTI amosite sample still demonstrates the greatest potency on a per EMP basis, while the other three samples exhibit similar potencies. Taken together, these data suggest that particle number is not the primary metric to explain the observed relative pattern of the IL-8 response in HAECs dosed on an equal mass basis. In part, this is not surprising since choice of any specific particle number dose metric assumes that the selected particles are representative of the potency of the complex sample as a whole.

Recalculation of the IL-8 transcript data as a function of TSA as measured by gas adsorption techniques (Figure 6A) results in a greater potency once again for the RTI amosite sample on a per unit surface area basis with the LA2000 sample exhibiting the least potency; however the difference in potency between the samples was significantly reduced. Recalculation of the IL-8 data with respect to the surface area of EMP with AR ≥ 3:1 only as measured by TEM resulted in even further reduction of the differential response between the RTI amosite sample and the other amphibole samples (Figure 6B) suggesting that elongated mineral particles are more significant contributors to the pro-inflammatory response than non-elongated particles.

Expression of the IL-8 data with respect to the sum of the particle lengths for all particles (Figure 7A) and for EMP only (Figure 7B) results in the generation of comparable plots due to the fact that the shorter particles, which are excluded in the EMP-only plot, are not weighted as much as the longer particles that are included in both plots. Comparison of the surface area dose metric plots and sum of the lengths dose metric plots for EMP only (Figure 6B and 7B) show many similarities which is to be expected since particle length is the principal dimension contributing to the calculated surface area.

### Surface reactivity

**Figure 8:**
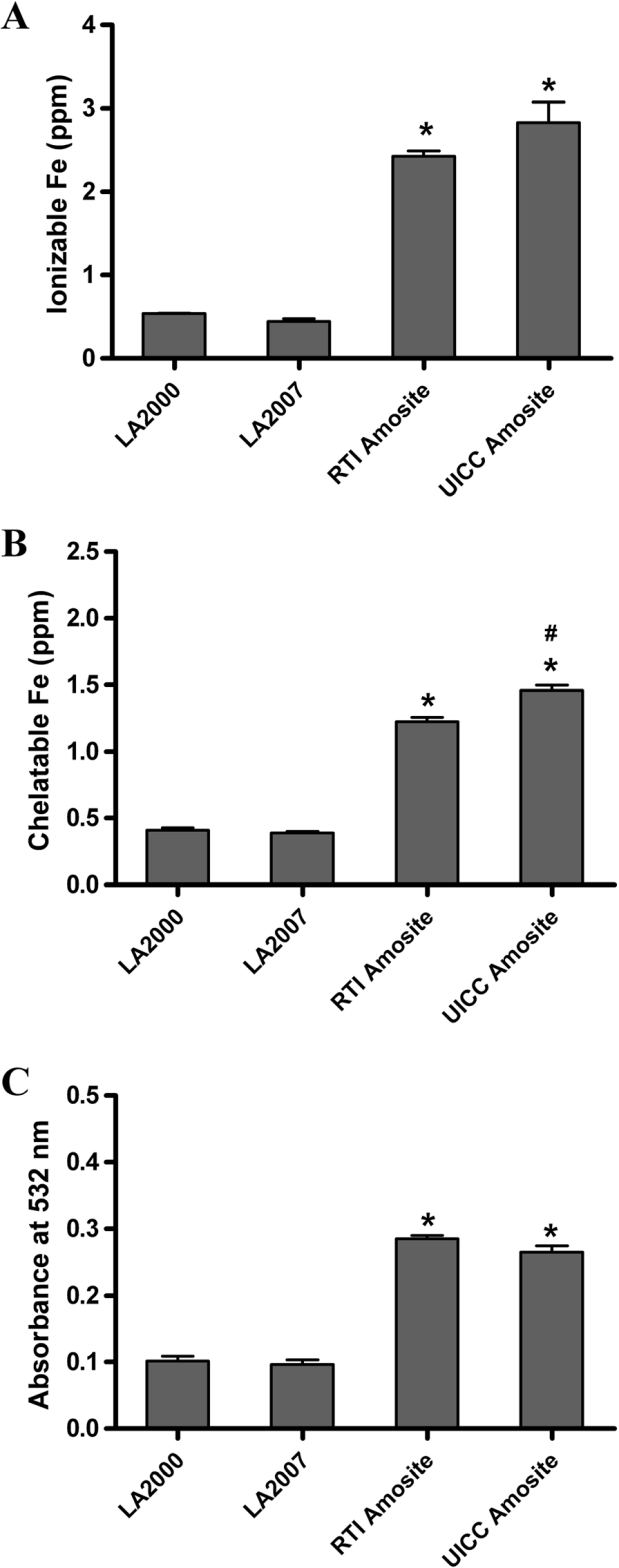
**A cellular determination of surface iron concentrations and corresponding reactive oxygen species generation by each of the asbestos mineral types. (A)** Ionizable iron concentrations measured by ICP-OES following treatment of the samples with 3.0 N HCl **(B)** Surface chelatable iron concentrations measured by ICP-OES following treatment of the samples with citrate-bicarbonate-dithionite (CBD) **(C)** Spectrophotometric assay measuring the thiobarbituric acid-reactive products of deoxyribose as a measure of hydroxyl radical production. Significant difference between the LA and amosite samples are denoted with (*). Significant difference between the two LA samples or between the two amosite samples denoted with (#). Differences are considered significant if *p* < 0.05.

One theory for the inherent toxicity associated with asbestos fibers involves free radical production catalyzed by surface-associated iron via Fenton chemistry [24-27]. Therefore, we quantified surface iron concentrations as well as surface reactivity with respect to hydroxyl radical production for all four samples to assess the effect of these two properties on the IL-8 response in HAEC. Surface iron was quantified by two complementary techniques: the first measured the amount of ionizable iron following treatment of the samples with 3 N HCl and the second measured the amount of surface chelatable iron after treatment of the samples with a citrate-bicarbonate-dithionate (CBD) mixture. The concentration of ionizable iron did not differ significantly between the two LA samples or between the two amosite standard reference samples (*p* > 0.05) (Figure 8A); however both of the amosite reference samples had significantly greater ionizable iron concentrations compared with the two LA samples (*p* < 0.05). A similar trend was observed for the measurement of chelatable surface iron except that the UICC amosite sample had slightly greater chelatable iron concentration than the RTI amosite sample (Figure 8B). Since the RTI sample induces a more robust IL-8 response than the UICC sample, it does not appear that surface iron alone can account for the pro-inflammatory response.

Surface reactivity was also assessed by indirectly monitoring the production of hydroxyl radicals by the mineral samples using a colorimetric assay that measures thiobarbituric acid (TBA)-reactive products of deoxyribose. No difference in hydroxyl radical production was detected between the two LA samples or between the two amosite reference samples (*p* > 0.05) (Figure 8C). The relative quantities of hydroxyl radical produced by each of the amphibole samples followed a similar trend as observed in Figures 8A and B in that both of the amosite samples produced greater quantities of reactive oxygen species compared with the two LA samples suggesting a correlation between these parameters. However, there appears to be a lack of agreement between the amphibole surface area of these samples and their ability to produce reactive oxygen species (ROS). Furthermore, the observed trend in surface iron and surface reactivity with respect to ROS production does not follow the observed trend in IL-8 response (Figure 4) suggesting that although production of ROS is likely to be a contributing factor to the toxicity of these amphibole minerals it is clearly not the major determinant.

## Discussion

In the present study, primary human airway epithelial cells were exposed on an equal mass basis to four amphibole samples: LA2000, LA2007, RTI amosite and UICC amosite. Messenger RNA transcript levels of four genes known to contribute to asbestos-induced inflammation [17] were quantified in response to each of the amphibole samples. When evaluated on a mass basis, all the asbestos samples induced significant increases in all four gene products. However, the RTI amosite was the most potent at inducing pro-inflammatory mediator expression in HAEC relative to the other three samples.

Selecting the appropriate metric by which to normalize the dose received in cellular exposures to mineral particles *in vitro* is a critical yet controversial issue [28]. Historically, mass has been the most commonly utilized metric in comparative studies of particulates [14,17,29-33]; however without additional information on the physicochemical properties of the samples under investigation there is a lot of uncertainty with this approach that makes interpretation of comparative toxicity data difficult. To address the issue of alternative dose metrics, we have, for the first time, conducted extensive physicochemical characterization of the Libby amphibole samples, in addition to the more well-studied standard reference amosite samples, to ensure accurate interpretation of the acquired cellular response data. Included in this comprehensive characterization were several parameters deemed to be fundamentally important to the mechanism of action of amphibole fibers including particle size distribution, particle number, surface area, and surface properties such as surface-conjugated iron ions and production of reactive oxygen species by the mineral particles. Furthermore, the effect of morphology on the IL-8 response was assessed in the current study by evaluating each dose metric as a function of all particles in the sample as well as a function of only the elongated mineral particles (EMP) with aspect ratio ≥ 3:1, which is the historical definition of a “fiber” when based on dimensional criteria.

When dosed on an equal mass basis, the RTI amosite sample induced the greatest IL-8 response and yet it was shown to contain significantly fewer particles per milligram of either total particles or EMP. Conversely, the LA2000 sample contained a substantially greater number of EMP yet generated the same IL-8 transcriptional response in HAEC as the LA2007 and UICC amosite samples suggesting that some other determinant besides particle number is driving the observed pro-inflammatory response. There is substantial evidence that suggests that surface characteristics of asbestos particles are correlated with their toxicity [24-27]. Consequently, surface area has become an important alternative dose metric to mass for comparative studies. The most widely utilized method for measuring surface area is based on a gas adsorption technique [34]. This method measures the number of gas molecules adsorbed onto the surface of the particles resulting in a high degree of accuracy due to the ability of the gas molecules to traverse into crevices present on the particle surface or into fiber bundles. However a limitation of this technique is that it does not discriminate between amphibole particles and non-amphibole accessory minerals or particles with fibrous and non-fibrous dimensions. Despite this caveat, the ease and relative low cost of this technique still makes it favorable for use as a tool to measure surface area for the purposes of comparative toxicity. Conversely, the use of electron microscopy for the calculation of surface area allows for the select determination of the surface area of the specific groups of particles only, such as elongated mineral particles, that may be more relevant to the toxicological assessment. Although this method is not as precise in detecting surface irregularities on each particle, it provides a fairly accurate approximation of particle surface area.

Recalculation of the IL-8 transcriptional data with respect to surface area resulted in a significant mitigation of the differential response between the RTI amosite sample and the other three samples. This result was most notable when only EMP were included in the surface area calculation, suggesting that not only is surface area an important determinant when assessing fiber toxicity, but that the contribution by the elongated minerals is more significant than the non-elongated particles. This finding is consistent with those reported by Timbrell, et al in which the authors explored different physical properties as alternative dose metrics to explain the degree of fibrosis observed in the post-mortem lungs of mine workers exposed to varying amphibole fiber types [35]. Although metrics including mass concentration and fiber number concentration were considered in this report, the data supported the conclusion that “asbestosis-producing ability is independent of amphibole type but depends on the total surface area of long resident fibers per unit weight of lung tissue”. The fact that the present study came to the same conclusion using an *in vitro* exposure system in which an acute pro-inflammatory response was quantified as a model for the early initiating events for fibrosis provides support for the use of *in vitro* exposure methods to inform toxicological assessments related to unknown elongated mineral particles. Although surface area appears to be an important metric, the amphibole-specific surface property responsible for inducing the pro-inflammatory response in HAEC does not appear to be solely due to the amount of surface-conjugated iron ions or reactive oxygen species production by the mineral particles since these properties did not correlate with the surface area values observed for the amphibole samples.

To assess the contribution of fiber length to the pathogenic potential of the amphibole fibers the sum of the particle lengths was calculated as opposed to applying an arbitrary filter at a specific length cutoff. By taking this approach, length is treated as a continuum as it relates to toxicological potential such that longer fibers are weighted more heavily than the shorter fibers. In terms of IL-8 transcript induction, this metric was also found to eliminate the difference in potency between the RTI amosite sample and the other samples, which is not surprising since surface area and sum of the lengths were found to be highly correlated. The importance of fiber length as a determinant for inducing a pro-inflammatory response in HAEC is in accordance with our previous report on the size-fractionated PM_2.5_ samples of LA2000 and RTI amosite (LA_2.5_ and AM_2.5_, respectively), which were equally pure amphibole samples and demonstrated comparable total surface area values; however the AM_2.5_ sample possessed considerably longer fibers as opposed to the LA_2.5_ sample and induced a 3-fold greater IL-8 transcriptional response [17]. This hierarchy of potency between the RTI amosite and LA samples observed on an equal mass basis was also consistent with the findings of the animal toxicology studies reported as part of the Libby Action Plan in which rats were dosed by single intratracheal instillation [31]. The concept of particle length as a critical determinant for asbestos toxicity is of course hardly a novel concept as numerous reports have shown correlations between fiber length and carcinogenic outcomes [28,36] as well as on lung injury and inflammation through incomplete phagocytosis of elongated fibers by alveolar macrophages [37]. However, the effect of particle length on the acute pro-inflammatory response in airway epithelial cells is not well established. This report supports the notion that the sum of particle lengths and surface area, especially for those particles with AR ≥ 3:1, are critical determinants for the induction of a pro-inflammatory response in airway epithelial cells.

The current study focused on the IL-8 response in HAEC as a biomarker of inflammation that can be linked with asbestos-induced inflammation observed in parallel animal studies done using these particles [31,38]. Future studies investigating other chemokines and cytokines as well as additional markers of apoptosis, cell survival and DNA damage may shed even more light on the critical determinants that drive different aspects of cellular toxicity in response to asbestos exposure. Furthermore, evaluating the cellular responses of other cell types including type I and II alveolar epithelial cells and alveolar macrophages would further contribute to our understanding of the molecular mechanisms of asbestos-related lung diseases.

## Conclusion

Amphibole mineral particles collected from the decommissioned mine in Libby, MT are shown in this study to be as potent at inducing a pro-inflammatory response in lung epithelial cells as UICC amosite but are less potent than the RTI amosite sample when dosed on an equal whole sample mass basis. Expression of the data with respect to surface area or sum of particle lengths of elongated mineral particles with AR ≥ 3:1 normalizes this difference in response suggesting that doses described by these two metrics, which are correlated, contribute significantly to the pathogenic potential of these amphibole fibers with respect to inducing a pro-inflammatory response in airway epithelial cells.

## Methods

### Amphibole samples

Libby amphibole (LA) was collected from the Rainy Creek Complex near Libby, Montana in the year 2000 (LA2000) or in the year 2007 (LA2007) and processed by U.S. Geological Survey (USGS, Denver, CO, USA) as described in their recent report [23]. A standard reference sample of amosite was obtained from RTI International (Research Triangle Park, NC, USA), and is referred to herein as RTI amosite. Additionally, the more commonly used standard reference sample of amosite, Union for International Cancer Control amosite (UICC amosite), was also included in this study as an additional comparative reference sample.

Scanning electron microscopy (SEM) images and energy-dispersive x-ray spectra (EDS) were acquired for all samples. Platinum-coated specimens were examined in a scanning electron microscope (JEOL 6400 SEM, JEOL USA, Inc.) equipped with a 30-mm^2^ energy-dispersive x-ray detector, pulse processor, power supply, and analyzer (Link 2000, Oxford Analytical Systems, High Wycombe, UK) with interface to an electronic multichannel analyzer (Spectral Engine, 4pi, Inc., Durham, NC, USA) and microcomputer (Macintosh Quadra 650, Apple Computer, Cupertino, CA, USA). An area was selected for analysis, and secondary electron images were obtained for reference.

Sample characterizations were obtained for the LA2000, LA2007, RTI amosite and UICC amosite samples including measurement of the particle size distribution (length, width and aspect ratio) by counting approximately 500 particles using transmission electron microscopy (TEM) coupled with selected area electron diffraction (SAED) and energy-dispersive x-ray spectroscopy (EDS). Sample analyses were conducted by EMSL Analytical, Inc. (Libby, MT, USA) and a full description of each particle is detailed in Additional file 1: Tables S1-S4. All objects were included in the particle count if they had a length of at least 0.2 μm. For the purposes of this paper, “total particles” refers to any mineral with L ≥ 0.2 μm with no aspect ratio restrictions whereas “elongated mineral particle (EMP)” specifically refers to those particles that have an aspect ratio of ≥ 3:1. Particles per milligram of sample were calculated using the TEM analytical data by dividing the number of particles counted by the weight of the counted particles.

Sum of the lengths dose was calculated by summing the lengths of the all particles or EMP only and expressing this value with respect to the mass of sample counted on the filter in units of m/μg. Reported ∑ L doses were further mass adjusted by dividing each value by the respective ratio of the calculated mass of the counted particles determined using particle dimensions [L*W*T*ρ (μg)] relative to the mass of the sample counted on the filter [m_filter_ (μg) = filter loading (μg/mm^2^) × area counted (mm^2^)].

### Surface area of amphibole samples

Total surface area (TSA) was measured by krypton gas adsorption using Brunauer-Emmet-Teller (BET) theory (Micromeritics Analytical Services, Norcross, GA, USA) for all samples. Reported TSA values are an average of two replicate measurements. Additionally, the surface area of the elongated mineral particles (EMP) only (AR ≥ 3:1) was calculated using particle measurements acquired by TEM (EMSL Analytical, Inc.). Surface area per mass of each EMP was calculated using the equations [2LW + 2LT + 2WT] ∕ [LWTHρ], respectively where ρ is the density of the amphibole particles (3.15 g/cm^3^ for LA and 3.40 g/cm^3^ for amosite). Thickness (T) values were calculated using W/T ratios of 3.15 for LA particles with AR ≥ 3:1, 3.00 for LA particles with AR < 3:1, 3.40 for amosite particles with AR ≥ 3:1, 3.00 for amosite particles with AR < 3:1 and 2.00 for non-amphibole particles as determined from previously acquired size distribution data (Cook, unpublished), and confirmed in the current study through a secondary TEM analysis of ~100 amphibole particles. Amphibole identification was established based on observation of a layer line spacing of approximately 5.3 Å in the SAED pattern and the chemical composition as determined by EDS [39]. In this secondary analysis, T was determined using a shadow casting technique with 0.1 μm and 0.5 μm polystyrene beads as reference. Reported EMP surface areas were mass adjusted by dividing each SA value by the respective ratio of the calculated mass of the counted particles determined using particle dimensions [L*W*T*ρ (μg)] relative to the mass of the sample counted on the filter [m_filter_ (μg) = filter loading (μg/mm^2^) × area counted (mm^2^)].

### Measurement of ionizable and chelatable surface iron concentrations

Ionizable iron concentrations associated with each of the amphibole samples were quantified in an acellular assay [40,41]. One mg of each sample was suspended in 1.0 mL of 3.0 N HCl and agitated in a water bath at 70°C for 60 min. After centrifugation at 1500 *g* for 10 min, iron in the supernatant was assayed using inductively coupled plasma optical emission spectroscopy (ICP-OES; Model Optima 4300D, Perkin Elmer, Norwalk, CT, USA) operated at a wavelength of 238.204 nm. Ionizable iron was measured in triplicate and repeated once.

Concentrations of surface-chelatable iron were measured using citrate-bicarbonate-dithionite (CBD) methodology. Surface-complexed ferric ions are reduced to ferrous ions which are then able to be chelated by the citrate ligand and subsequently assayed in the reaction supernatant. One mg of each of the four amphibole samples was exposed to 0.3 M sodium citrate, 1.0 M sodium bicarbonate, and 10 mg sodium dithionite. This suspension was agitated in a water bath at 70°C for 30 min and centrifuged at 1500 *g* for 10 min, and the supernatant was assayed for iron using ICP-OES. Surface iron was quantified in triplicate and the experiment repeated once.

### Oxidant generation measured by TBA-reactive products of deoxyribose

Oxidant generation by each of the four amphibole samples was measured via thiobarbituric acid (TBA)-reactive products of deoxyribose as previously described [42]. Briefly, 2-deoxy-D-ribose reacts with an unspecified iron-catalyzed oxidant(s), to form a mixture of malondialdehyde adduct species. After heating with TBA at a low pH, a pink chromogen indistinguishable from a TBA-malondialdehyde adduct is generated, which can be used as an assay for hydroxyl radical production by measuring its absorbance at 532 nm. Measurements were done in triplicate and repeated twice.

### Cultured cells

Primary human airway epithelial cells (HAECs) were obtained by brush biopsy of the mainstem bronchus of healthy, non-smoking adult volunteers undergoing routine fiber-optic bronchoscopy for the purpose of sample collection for scientific study. The human subject protocol under which these cells were obtained was reviewed and approved by the Human Subjects Institutional Review Board at the University of North Carolina at Chapel Hill as well as the US EPA. HAECs were grown on Corning Costar plastic tissue culture plates (Corning, Inc. Wilkes-Barre, PA, USA) in supplemented bronchial epithelial cell growth medium (BEGM) (Clonetics, San Diego, CA, USA), as described previously [43]. Briefly, the cells collected on the brush were plated on plastic tissue culture plates and grown and expanded submerged under media until exposure to the amphibole particles at passage 3.

### In vitro exposures for dose–response comparison

All amphibole samples were prepared fresh as 2 mg/ml stock solutions in sterile water and vigorously vortexed for particle dispersion. HAECs were exposed as a confluent monolayer to 0 μg/cm^2^ (vehicle-only control) or to 2.64, 13.2, or 26.4 μg/cm^2^ (10, 50, or 100 μg/mL) of each of the amphibole samples for 24 h. This time point was chosen based on previous work from our laboratory that showed maximal response in mRNA transcript levels of key pro-inflammatory genes, including IL-8, after a 24 h exposure as compared to a 2 h exposure, which showed minimal increases in these same transcripts [17]. The dose response was repeated with three biological replicates in which cells from three different volunteers were used (*n* = 3). Triplicate exposure replicates were conducted within each biological experimental replicate for a total of *n* = 9 for each exposure condition. Following the exposure, the cells were washed two times with 1x phosphate-buffered saline (PBS) (GibcoBRL, Gaithersburg, MD, USA), lysed by the addition of guanidine isothiocyanate-based lysis solution (RLT buffer supplied with Qiagen RNeasy Minikit, Valencia, CA, USA), and dislodged from the plate with a cell scraper. The cell lysates were sheared three to five times with a 1 cc syringe and 21-guage needle and the lysates stored at -80°C until processed for RNA isolation.

### Cytotoxicity studies

Supernatants collected from treatment and control wells after a 24 h exposure to the maximum concentration of each sample were analyzed for cellular release of lactate dehydrogenase (LDH) protein using a Cytotox 96 Non-Radioactive Cytotoxicity Assay kit (Promega, Madison, WI, USA) per the manufacturer’s instructions. LDH concentrations were expressed as a percentage of LDH detected in the cell culture medium relative to the total LDH present in the cell lysates and supernatant combined.

### Quantitative RT-PCR

Dose-dependent relative mRNA transcript changes of the pro-inflammatory markers IL-8, IL-6, TNF, and COX2 in HAECs were quantified using qRT-PCR following a 24 h exposure to the various amphibole samples. Total RNA was isolated using a Qiagen RNeasy Mini Kit according to manufacturer’s instructions (Qiagen, Valencia, CA, USA) and 100 ng was reverse transcribed to generate cDNA using the High Capacity cDNA Reverse Transcription kit (Applied Biosystems, Foster City, CA, USA). Quantitative fluorogenic amplification of cDNA was performed using the ABI StepOnePlus^TM^ Real-Time PCR System (Applied Biosystems) with TaqMan Universal PCR Master Mix using primer/probe sets described earlier [17]. 5′ FAM/3′ TAMRA) CCTTGGCAAAACTGCACCTTCACACA) multiplexed with β-actin (*ACTB*) primer/probe sets was the normalizing housekeeping gene (20x VIC/MGB, Primer Limited, Applied Biosystems). C_t_ values from triplicate technical replicates for each sample were averaged and data analyzed using the 2^-ΔΔCt^ method to obtain fold change values with respect to the vehicle-only control. The resultant data from each biological replicate were combined to give the average fold changes ± SEM. Given that inherent variability is expected for the observed responses between primary cells obtained from different biological donors, the variance (as indicated by the error bars) observed in the present study for the IL-8 response is comparable to those reported by us and others in this cell type in response to particle exposure [17,20].

### Statistical analysis

Data are presented as mean ± SEM. Quantitative RT-PCR data for IL-8 were evaluated by one-way ANOVA with Dunnett post hoc procedure for comparison between treatment and control cells or two-way ANOVA with Bonferonni post test for comparison between different amphibole types. Statistical analysis was not conducted on the re-analyzed IL-8 data for the different dose metrics (shown in Figures 5, 6 and 7) since neither dose nor IL-8 response was controlled for in these re-analyses. Surface iron, water soluble iron and oxidant generation data were evaluated by one-way ANOVA with Tukey’s post test. Significance was assumed at *p* < 0.05.

## Competing interests

The authors declare that they have no competing interests.

## Authors’ contributions

KED participated in the design of the study, conducted the exposures, analyzed the data and drafted the manuscript. PMC and SHG aided in the interpretation of the TEM data and helped draft the manuscript. LAD conducted the cell culture and helped with the exposures. RKM conducted the TEM analysis of the samples. AJG conducted the assays for surface iron and surface reactivity. VLR acquired the SEM images of the samples and EDS spectra. RBD conceived of the study, participated in its design and helped to interpret the data and draft the manuscript. All authors read and approved the final manuscript.

## Supplementary Material

Additional file 1: Table S1 Particle size distribution of the particles in the LA2000 sample. **Table S2.** Particle size distribution of the particles in the LA2007 sample. **Table S3.** Particle size distribution of the particles in the RTI amosite sample. **Table S4.** Particle size distribution of the particles in the UICC amosite sample. Click here for file
